# High expression of miR-222-3p in children with Mycoplasma pneumoniae pneumonia

**DOI:** 10.1186/s13052-019-0750-7

**Published:** 2019-12-16

**Authors:** Chu Chu, Xiaoli Lei, Yuqin Li, Yali Luo, Ying Ding, Weifang Zhou, Wei Ji

**Affiliations:** 1 Department of Infectious Disease, Children’s Hospital of Soochow University, Soochow University, Suzhou, 215123 China; 2 Department of Respiratory Disease, Children’s Hospital of Soochow University, Soochow University, Suzhou, 215123 China

**Keywords:** CD4, Children, MiR-222-3p, Mycoplasma pneumoniae pneumonia

## Abstract

**Objectives:**

Mycoplasma pneumoniae is a leading cause of community-acquired pneumonia in children. However, its mechanism of pathogenesis is not fully understood, and microRNAs might play a role. This study aimed to explore the microRNA-222-3p (miR-222-3p) expression and its possible role in children with M.pneumoniae pneumonia (MPP).

**Methods:**

Thirty-six children with MPP and twenty-seven age-matched controls from Children’s Hospital of Soochow University were enrolled in this study. MiR-222-3p and cluster of differentiation 4 (CD4) mRNA were detected using real-time PCR in children’s peripheral blood plasma samples. THP-1 cells and mice were stimulated with M.pneumoniae lipid-associated membrane proteins(LAMPs).

**Results:**

Children with MPP had significantly higher levels of miR-222-3p and lower levels of CD4 in peripheral blood plasma (*P* <  0.05). Additionally, Sixteen children with MPP complicated with pleural effusion had higher miR-222-3p levels than those without pleural effusion. MiR-222-3p or CD4 in THP-1 cells increased or decreased, respectively, in a dose dependent manner after LAMP stimulation. In LAMP-stimulated mice massive inflammatory cells infiltrates surrounded the bronchioles, and miR-222-3p increased in the bronchoalveolar lavage fluid. In conclusion, miR-222-3p was highly expressed in children with MPP, especially those with pleural effusion.

**Conclusion:**

Small sample studies showed that M.pneumoniae or its LAMPs could increase miR-222-3p and decrease CD4 in macrophages,both in vitro and vivo.Thus, miR-222-3p might be an MPP biomarker for the diagnosis and prognosis.

## Introduction

Mycoplasma pneumoniae is an important cause of community-acquired pneumonia (CAP) in children and young adolescents, accounting for 20 to 40% of CAP in children [1]. Some pathogenic factors of M. pneumoniae, such as hydrogen peroxide, Community-Acquired Respiratory Distress Syndrome (CARDS) toxin, nuclease and lipid-associated membrane proteins (LAMPs), have been reported to be associated with the development of pneumonia [2–5]. However, these pathogenic factors are insufficient to explain the pathogenic mechanism of *M. pneumoniae* pneumonia (MPP) in children.

Nowadays, in the post-genome era, RNAomics (including mRNA, miRNA, piRNA, lncRNA, etc.) has become an important research hotspot.In addition to protein-coding genes, non-coding RNAs (such as miRNAs, lncRNAs) are equally important in life activities such as gene regulation and cell development, regulating genes at multiple levels and exerting important biological functions.A microRNA (miRNA) is an endogenous single-stranded small molecule RNA produced by a non-coding region of the genome. Studies have found that miRNA expression abnormalities are closely related to the occurrence and development of diseases, and some miRNAs are associated with tumor cell proliferation, drug resistance and prognosis [6].Down-regulation of the expression of such miRNAs can inhibit the proliferation of tumor cells, accelerate apoptosis, and bring new ideas for cancer treatment.In the early stage of the experiment, the expression profiles of miRNAs in peripheral blood of children in MPP group and NC group were compared, and 26 differentially expressed miRNAs, including miRNA-222-3p, were up-regulated, including miRNA-5704. The down-regulation of 7 miRNAs suggests that miRNAs participate in the pathological process of MPP and participate in the immune response of Mycoplasma pneumoniae. At present, few studies have found that mir-222-3p has high expression in children’s mpp, therefore the miR-222-3p with up-regulated expression in the gene chip was selected as the research object. Target gene prediction analysis of miR-222-3p was performed by Targetscan, Pictar, and miRDB databases to determine that CD4 molecule is a potential target gene of miRNA-222-3p.

## Materials and methods

### Patient characteristics

36 children with MPP from Children’s Hospital of Soochow University were enrolled in this study from March 2014 to June 2015, and immunodeficiency, premature delivery, recurrent pneumonia and recent use of immunosuppressive agents and immunomodulators were excluded.There were twenty males and sixteen females, with an average age of (6.74 ± 2.82) years. Among them, sixteen children with pleural effusion. Twenty-seven healthy children in the same period served as control group, fifteen males and twelve females, with an average age of (7.08 ± 2.74) years. There was no difference in sex and age between the two groups (*p* <  0.05), which was comparable.The inclusion criteria of the control group were no history of immune system diseases and chronic infectious diseases and no respiratory tract infections, other acute and chronic diseases, no allergies and diseases closely related to immunity in the past two weeks. Ethical approval for the study was received from the Institutional Medical Ethics Review Board of Children’s Hospital of Soochow University, and the study was performed in accordance with the Declaration of Helsinki. The diagnosis of *M. pneumoniae* infection was based on serologic testing and confirmed by polymerase chain reaction (PCR) tests of nasopharyngeal secretions. Diagnosis in all patients was made based on clinical and radiological signs of CAP in all patients according to British Thoracic Society Guidelines for the Management of Community Acquired Pneumonia [7]. Upon patient admission to the hospital, their pediatricians completed a questionnaire regarding the patient demographic and clinical data. Meanwhile, peripheral blood samples were obtained for use in miR-222-3p and CD4 detection. Twenty-seven of age-matched controls who had not experienced any acute respiratory infections within the previous four weeks were chosen randomly from surgery wards.

Peripheral blood was collected for laboratory examination and for miR-222-3p and CD4 mRNA detection. Laboratory data were also collected, such as white blood cell count (WBC), absolute neutrophil count, C-reactive protein (CRP), lactate dehydrogenase (LDH), and lymphocytes subgroups.

### Plasma samples and quality control

Peripheral blood samples from three healthy controls and three MPP cases were obtained and then drawn into EDTA-containing glass tubes. Samples were immediately centrifuged at 3500×*g* for 15 min at 4 °C. The resulting plasma samples were then stored at − 80 °C until analysis. To determine the levels of free haemoglobin in the samples, 1.5 μl of total plasma was analyzed spectrophotometrically (NanoDrop 1000, Thermo Scientific) [8]. Absorbance levels above 0.2 at 414 nm were indicative of free haemoglobin and thereby a higher degree of haemolysis and such samples were excluded from further analysis.

### Microarray analysis of miRNA in plasma

Total RNA was isolated from human plasma [9]. A 200-μl sample of the plasma was transferred into a 1.5 ml tube and mixed with 750 μl of lysis mixture containing RNA lysis reagent (Trizol, Ambion, USA). Microarray hybridization, data generation, and normalization were performed by Kangchen Biological Engineering Co. Ltd. Using a human miRNA chip (miRCURY™, Exiqon, Denmark), which contains probes for 3100 miRNAs. Normalization was performed using quantile algorithm. MicroRNAs were considered to be differentially expressed if they produced a *p*-value of <  0.05 and a false discovery rate of ≤0.05. Differentially expressed miRNAs were also designated as up-regulated or down-regulated if the differrence in MPP children compared to controls was more than two-fold.

### Real-time quantitative PCR (qRT-PCR) for miR-222-3p and CD4

Total RNA was extracted using Trizol reagent (Invitrogen) according to the manufacturer’s protocol. Next, 1 μg of total RNA was subjected to reverse transcription PCR using the TaqMan MicroRNA Reverse Transcription kit (Applied Biosystems) according to manufacturer’s protocol. The thermocycling conditions were: 60 min at 42 °C, followed by 15 min at 70 °C, and 30 min at 4 °C. qRT-PCR was performed using a TaqMan Universal PCR Master Mix kit (Applied Biosystems) in an Bio-Rad iQ5 Real-Time PCR System and U6 or β-action was used as an endogenous control for miR-222-3p and CD4 respectively. Reactions were performed in triplicate. The thermocycling conditions for amplifying miR-222-3p were: 95 °C for 30 s, followed by 45 cycles of 5 s at 95 °C and 60 °C for 30 s, and concluded with 1 min at 60 °C. Meanwhile, the thermocycling conditions for amplifying CD4 were: 95 °C for 30 s, followed by 45 cycles of 5 s at 95 °C and 57 °C for 20 s, and concluded with 1 min at 60 °C [10]. After finalization of the qRT-PCR experiments, the average values of the cycle threshold (Ct) of the reactions in triplicate were determined. Data analysis was performedusing the 2^-ΔΔ^Ct method.

### Extraction of LAMPs from Mycoplasma

The *M.pneumoniae* standard strains M129 (Institute of Pathogenic Biology, Medical Faculty of Nanhua University) were cultured with PPLO broth medium (BD Biosciences) under 37 °C for 5 or 7 days. Once the color of the medium changed to orange or yellow, we transferred an adequate amount of bacteria liquid to new PPLO broth medium for amplification culture and subseqently collected new bacteria liquid when the medium color changed to orange or even yellow bu. was clear and not cloudy. The MP culture medium growing to logarithmic phase was centrifuged in a sterile centrifugal tube at 4 °C for 20 min at 14000 rpm. Triton X-114 was added. After mixing, the upper water phase was discarded and repeated, adding 2.5 times volume of anhydrous ethanol, overnight at − 20 °C, the supernatant was discarded, the PBS suspension was re-suspended, and the LAMPs suspension after low-speed centrifugation and supernatant was taken, sub-packed in EP tube, Finally, the LAMP concentration was detected by using an enhanced BCA Protein Assay kit (Beyotime Biotech).

### Expression of miR-222-3p and CD4 in LAMP-stimulated THP-1 cells

THP-1 cells with a density of 1 × 10^5^ cells/ml were cultured in RPMI 1640 medium (Life Technologies, Carlsbad, CA, USA) and then harvested and centrifuged after reaching exponential growth. The resulting cell pellet was suspended in serum-free medium, 1 × 10^6^ cells were distributed across a 24-well plate. Different concentrations of LAMPs (2 μg/ml, 4 μg/ml, 6 μg/ml) were added into the wells and the THP-1 cells were co-culture with LAMPs for 16 h; a control group was treated with phosphate buffered saline(PBS) instead of LAMPs. Then THP-1 cells were then harvested for miR-222-3p and CD4 detection by real-time PCR as described above.

### Expression of miR-222-3p in BALF from LAMP-stimulated mice

Eight-week-old male BABL/c mice were purchased from the Chinese Academy of Sciences. All animal studies were approved by the institutional animal care and use committee at Soochow University and complied with the animal welfare act. The methods applied in this study were carried out in accordance with the approved guidelines. Briefly, mice were randomly divided into two groups (*n* = 5 per group): 1) control group treated with saline, 2) LAMP group stimulated by LAMPs. Each mouse was intranasal instillated with 50 μg of LAMPs or saline. The experiment was terminated at 48 h after LAMPs inhalation, and the lung tissues were harvested thereafter. This in vivo study was carried out in two separate experiments. BALF samples and lung tissues were harvested using a previously described method [11]. The collected fluid was then centrifuged at 500×*g* for 2 min at 4 °C. Trizol was added to the resulting pellet, which was then stored at − 80 °C, then harvested for miR-222-3p and CD4 detection by real-time PCR as described above. Meanwhile, the lung was fixed in 4% paraformaldehyde in PBS and embedded in paraffin. Lung sections of 4 μm in size were stained with hematoxylin and eosin for morphological evaluation.

## Results

### Demographic data and clinical characteristics of children with MPP

A total of 36 cases with MPP and 27 control children with selective surgery were enrolled in the present study. As shown in Table 1, the amounts of absolute neutrophils, CRP, LDH, CD3^−^CD16^+^CD56^+^ NK cells and CD19^+^CD23^+^ B cells in children with MPP were all significantly increased compared with controls (all *p* <  0.05).

**Table 1:** Demographic data and clinical characteristics of children with MP

Parameters	Control (*n* = 27)	MPP (*n* = 36)	*P* value
Age (mean ± SD, year)	7.1 ± 2.7	6.7 ± 2.8	0.637
male (n, %)	15 (55.6)	22 (61.1)	0.261
White blood cell count (mean ± SD, × 10^9^/L)	7.0 ± 1.9	8.1 ± 4.1	0.154
Absulte neutrophils (mean ± SD, × 109/L)	0.6 ± 0.3	2.1 ± 1.0	< 0.001
C-reactive protein (25th–75th, mg/L)	0.06 (0.01–0.4)	11.3 (5.7–19.5)	< 0.001
lactate dehydrogenase (mean ± SD, U/L)	236.8 ± 42.2	362.1 ± 129.0	< 0.001
Lymphocytes subgroups			
CD3^+^ (mean ± SD, %)	69.8 ± 4.6	67.9 ± 9.2	0.295
CD3^+^CD4^+^ (mean ± SD, %)	36.3 ± 4.4	36.6 ± 7.7	0.848
CD3^+^CD8^+^ (mean ± SD, %)	25.0 ± 2.5	26.0 ± 5.6	0.336
CD4^+^/CD8^+^ (mean ± SD, %)	1.5 ± 0.3	1.5 ± 0.5	0.934
CD3^−^CD19^+^ (mean ± SD, %)	15.9 ± 4.4	17.6 ± 5.7	0.212
CD3^−^CD(16^+^ 56^+^) (mean ± SD, %)	17.3 ± 6.3	13.1 ± 6.4	0.012
CD19^+^CD23^+^ (mean ± SD, %)	5.8 ± 2.0	8.5 ± 3.8	< 0.001

### Total miRNAs profiling from microarray analysis

**Fig. 1:**
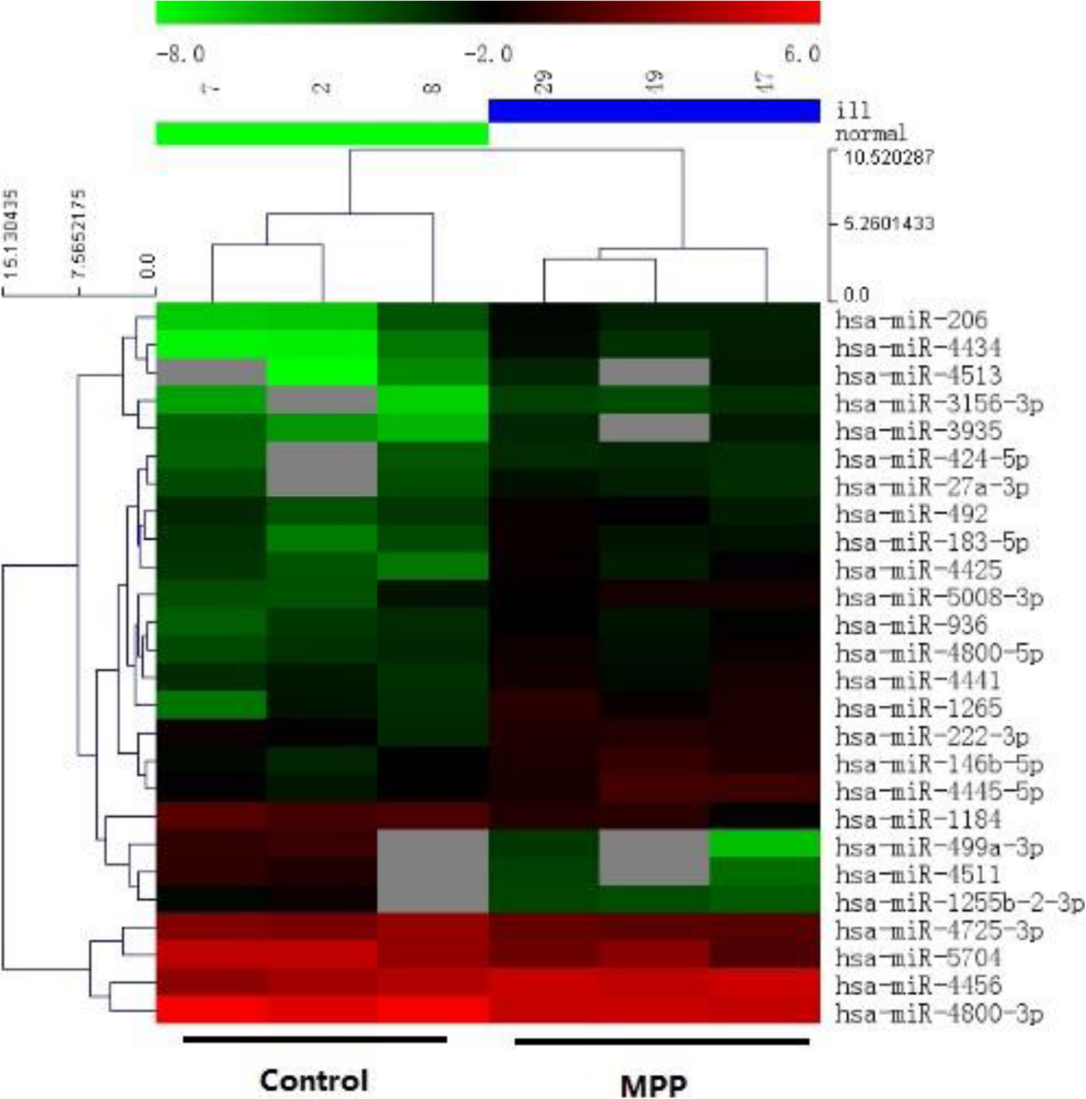
Heat map and cluster analysis of miRNA expression. Individual patient samples are shown in columns and miRNAs. Individual patient samples are shown in columns and miRNAs in rows. Of all differentially expressed miRNAs, 19 miRNAs were up-regulated including miR-222-3p and 7 miRNAs down-regulated

A heat map was generated from the microarray analysis of plasma from control children and those with MPP. Compared with the controls, there were 26 differentially expressed miRNAs with differences of > 2-fold in the plasma from children with MPP; Of these 19 miRNAs were up-regulated and 7 miRNAs were down-regulated (Fig. 1).

### Comparisons of miR-222-3p and CD4 levels between MPP cases and controls

**Fig. 2:**
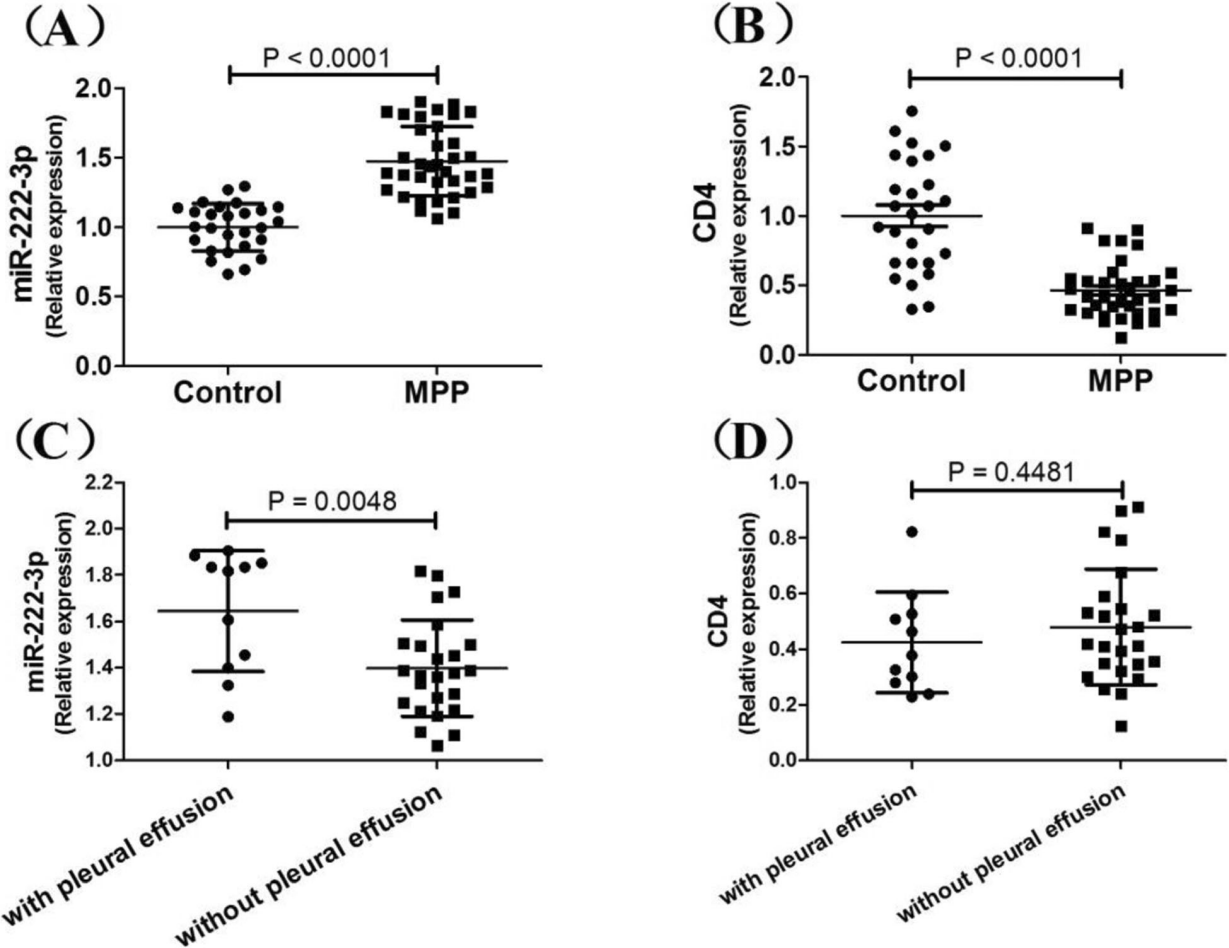
Comparisons of miR-222-3p and CD4 between MPP cases and controls. **a** miR-222-3p in PBMCs; **b** CD4 in PBMCs; **c** miR-222-3p in children with pleura effusion; **d** CD4 in children with pleural effusion. Error bars indicate standard error

According to the Target Scan 7.1 database, CD4 is one of the direct targets of miR-222-3p. Children with MPP had significantly higher levels of miR-222-3p in tneir peripheral blood mononuclear cells compared with controls (1.5 ± 0.2 vs. 1.0 ± 0.2, relative expression) and lower levels of CD4 mRNA (0.5 ± 0.2 vs. 1.0 ± 0.4, relative expression)(Fig. 2). Meanwhile, MPP cases with pleural effusion had higher levels of miR-222-3p compared with those that lacked pleural effusion; but no difference in CD4 mRNA levels was found between these groups(Fig. 2). No correlation was found between miR-222-3p and CD4 expression (*r* = − 0.231, *p* = 0.175).

### Expression of miR-222-3p and CD4 mRNA in LAMP-stimulated THP-1 cells

**Fig. 3:**
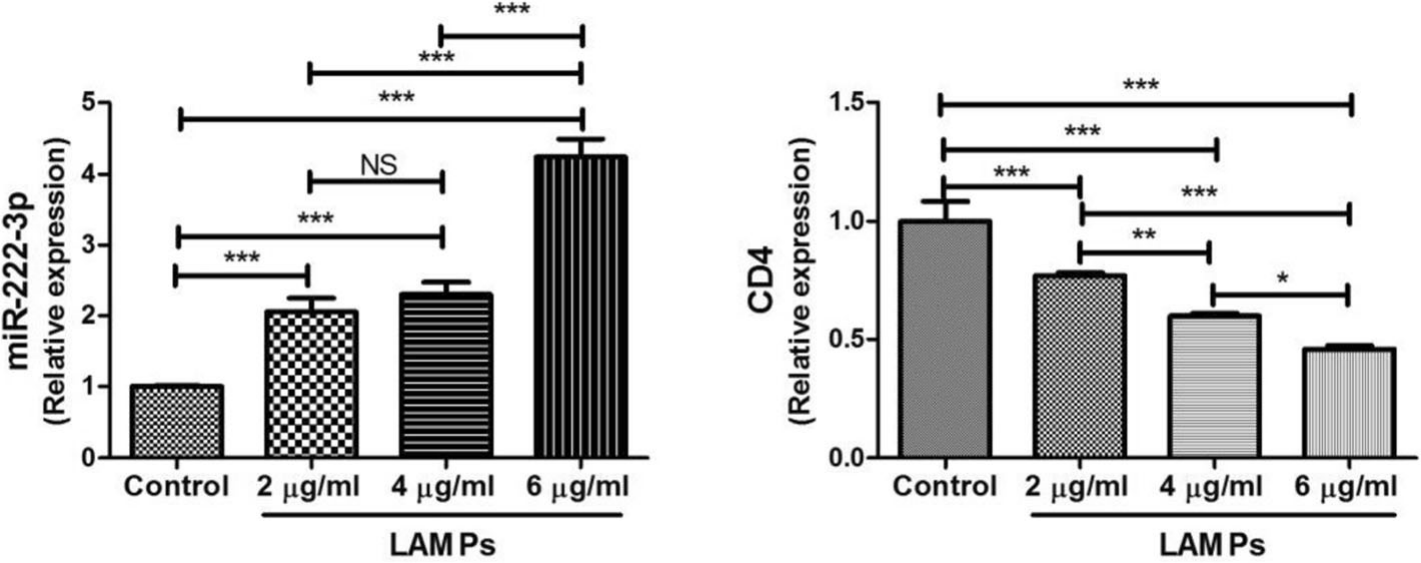
Expression of miR-222-3p and CD4 in THP-1 cells stimulated by LAMPs. THP-1 cells were adjusted into 1 × 10^6^/cell. Different concentrations of LAMPs (2 μg/ml, 4 μg/ml, 6 μg/ml) were added to co-culture with THP-1 cells and phosphate buffered saline as control. * *P* < 0.05; ** *P* < 0.01; *** *P* < 0.001. Error bars indicate standard error

**Fig. 4:**
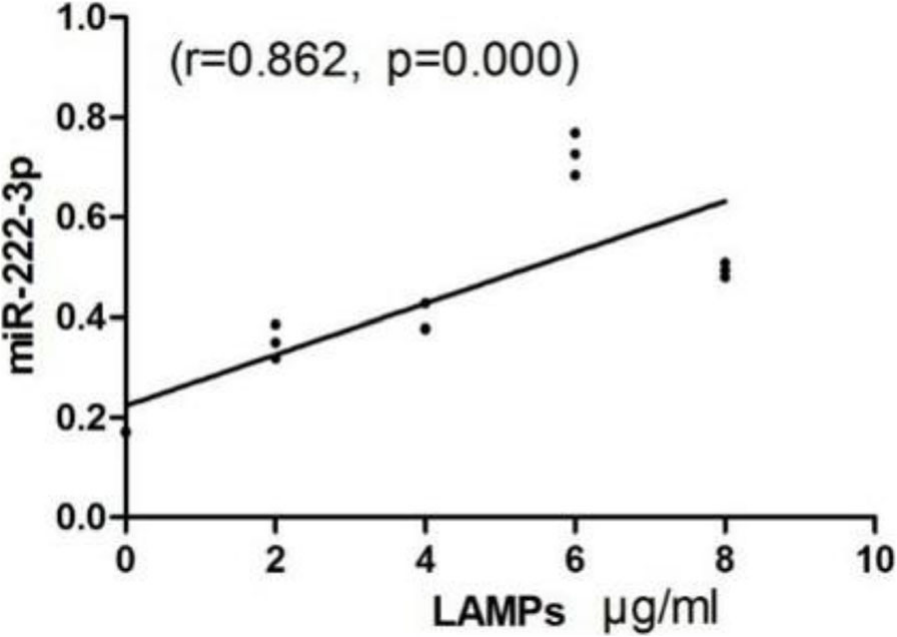
Correlation between LAMPs and miR-222-3p. A positive correlation was found between concentration of LAMPs and miR-222-3p in THP-1

Macrophages play a vital role in excessive inflammation caused by *M. pneumoniae* infection [12]. Based on this, we stimulated THP-1 cells with LAMPs from *M. pneumoniae* to explore the in vitro expression of miR-222-3p and CD4 mRNA in *M. pneumoniae*-stimulated monocyte cells. The level of miR-222-3p expressed by THP-1 cells increased in a dose-dependent manner after stimulation with LAMPs (Fig. 3). Furthmore, the miR-222-3p level was positively associated with the LAMP concentration (Fig. 4). In contrast, the CD4 level in THP-1 cells decreased in a dose-dependent manner after LAMP stimulation.

### Expression of miR-222-3p in the BALF of LAMP-stimulated mice

**Fig. 5:**
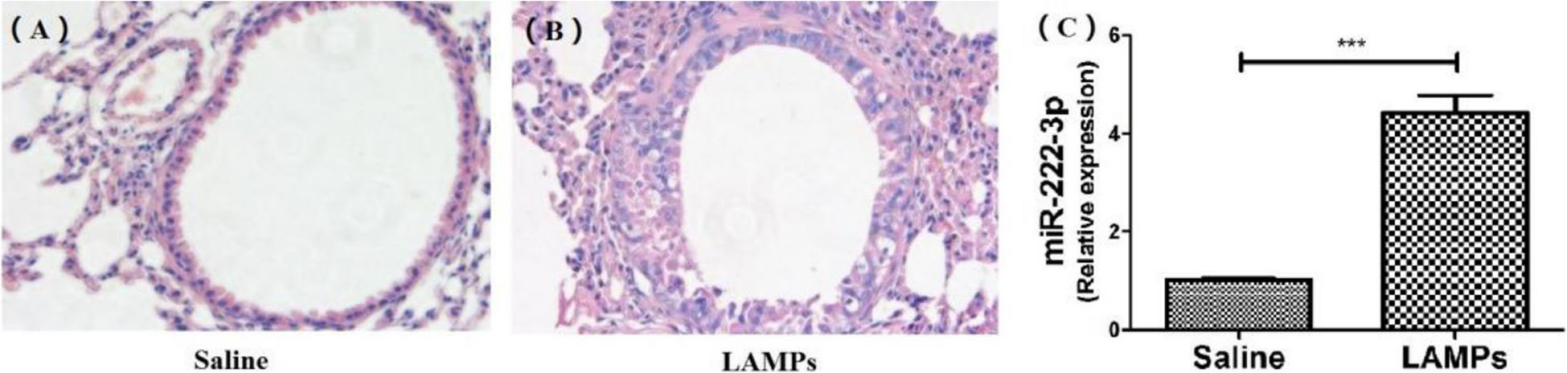
To determine the in vivo expression of miR-222-3p in BALF, LAMPs from M. pneumoniae were used to stimulate the lungs of BALB/c mice. Massive infiltrations of inflammatory cells were found around the bronchioles in LAMP-stimulated mice (**b**) compared with controls (**a**). Furthermore the level of miR-222-3p in the BALF was significantly higher for LAMP-stimulated mice than for saline-treated controls (**c**)

To determine the in vivo expression of miR-222-3p in BALF, LAMPs from M. pneumoniae were used to stimulate the lungs of BALB/c mice. Massive infiltrations of inflammatory cells were found around the bronchioles in LAMP-stimulated mice compared with controls (Fig. 5). Furthermore the level of miR-222-3p in the BALF was significantly higher for LAMP-stimulated mice than for saline-treated controls (Fig. 5) .

## Discussion

*M. pneumoniae* is a leading cause of CAP and excessive inflammation plays an important role in its disease severity [13]. However, the mechanism and pathology of the excessive inflammation is not fully understood. To the best of our knowledge, this is the first study to explore the miRNA expression in children with MPP. Here, we found that children with MPP had significantly higher levels of miR-222-3p and lower levels of CD4 mRNA in their peripheral blood mononuclear cells. Both in vitro and in vivo, miR-222-3p could be induced by stimulation with LAMPs from *M. pneumoniae*. Additionally, children with pleural effusion had higher miR-222-3p levels than those without pleural effusion.

Various cells are involved in the inflammation response, including macrophages, lymphocytes, bronchial epithelial cells, and neutrophils. Macrophages are essential for the clearance of *M. pneumoniae* from the lungs, and they play a vital role in *M. pneumoniae* infection [14]. Additionally, macrophages are heterogeneous immune cells with distinct origins, phenotypes, functions, and tissue localizations [15]. Their susceptibility to infection by microbes is subject to variations their differentiation and inflammation states, owing in part to regulatory miRNAs [16]. For example, miR-125a-3p promotes THP-1 macrophages differentiation and apoptosis by down-regulating NF1 and Bcl-2 [17]. MiR-125b and let7b can regulate macrophage cytokine production including TNF-α, IL-1β, IL-6, and IL-8 [18]. In addition, miR-155 and miR-146a form a complex network of cross-regulations to control gene transcription in macrophages for modulating inflammatory responses [19].

Previous studies of miR-222 in regulating macrophages has mostly focused on its role in tumors [20, 21]. Recently, Lodge found that CD4 is the direct target of miR-222 through luciferase assays and reported that the ectopic expression of miR-222 mimics in human macrophages reduced both CD4 mRNA and cell surface CD4, which resulted in virus infection [22]. Furthermore, the observed decrease of CD4 in TNF-α-treated macrophages is largely due to miR-222 induction [22]. In our previous study, we found that TNF-α level was significantly higher in M. pneumoniae pneumonia subjects [23]. Taken together, it hypothesized that M. pneumoniae could induce miR-222 expression in macrophages through LAMPs or pro-inflammatory cytokines such as TNF-α and high expression miR-222 subsequently decreases CD4 which is associated with macrophage function.

Some limitations of this study should be noted. Firstly, small sample size may result in study bias and it is necessary to increase sample size for verification of clinical outcomes. Secondly, CD4 as a target of miR-222-3p is predicted by TargetScan database and not confirmed by luciferase assays.

## Conclusion

Small sample studies showed that *M.pneumoniae* or its LAMPs could increase miR-222-3p and decrease CD4 in macrophages,both in vitro and vivo. Thus, miR-222-3p might be an MPP biomarker useful for diagnosis and prognosis. In future studies, larger samples will be collected to verify how highly expressed microrna-222-3p regulates the inflammatory expression of monocytes, thus playing a role in Mycoplasma pneumoniae in children.

## Data Availability

All data is available.

## Abbreviations

BALF: Bronchoalveolar lavage fluid
CAP: Community-acquired pneumonia
CARDS: Community-Acquired Respiratory Distress Syndrome
CD4: Cluster of differentiation 4
CRP: C-reactive protein
LAMPs: Lipid-associated membrane proteins
LDH: Lactate dehydrogenase
MiR-222-3p: MicroRNA-222-3p
MiRNAs: Micro RNAs
MPP: M. pneumoniae pneumonia
PCR: Polymerase chain reaction
qRT-PCR: Real-time quantitative PCR
WBC: White blood cells
